# Connectivity-Based Pain Recognition from fNIRS: Parsimonious Subject-Independent Classification

**DOI:** 10.3390/s26102947

**Published:** 2026-05-08

**Authors:** Mohammadreza Safari, Maryam Ghahramani, Raul Fernandez Rojas

**Affiliations:** BioSIS (Biosensing & Intelligent Systems) Lab, Centre for Intelligent Computing and Systems, University of Canberra, Bruce, ACT 2617, Australia; reza.safari6311@gmail.com (M.S.); maryam.ghahramani@canberra.edu.au (M.G.)

**Keywords:** pain assessment, fNIRS, functional and effective connectivity, machine learning, leave-one-subject-out, feature selection, model parsimony

## Abstract

Accurate pain assessment remains challenging, because pain is subjective, and common clinical tools rely on self-report or observation. Functional near-infrared spectroscopy (fNIRS) provides a non-invasive window into cortical haemodynamics and shows promise for objective pain assessment. In this study, we propose a subject-independent framework that models pain states using functional and effective connectivity features derived from fNIRS signals. Using the AI4PAIN dataset (65 participants, 24 channels), we extracted correlation, partial correlation, coherence, and Granger causality matrices from haemodynamic signals (HbO_2_, HHb, and HbT), yielding a comprehensive connectivity feature space. A three-class classification task (No Pain vs. Low Pain vs. High Pain) was evaluated using leave-one-subject-out (LOSO) cross-validation. Within each training fold, we standardised features and applied mutual-information-based feature selection; a systematic sweep showed that a reduced feature set of 700 connectivity features achieves performance comparable to the full 1380 features and constitutes the minimum configuration at which High Pain instances begin to be detected (High Pain recall ≈ 50%), whereas smaller feature sets produce near-zero High Pain recall. The proposed framework achieved best accuracy of 69.6%, with HHb achieving the highest accuracy (69.6%), followed by HbT and HbO_2_. Analysis of frequently selected features revealed a predominance of directed Granger-causality connections, indicating stable and interpretable connectivity patterns associated with pain processing. These findings demonstrate the feasibility of connectivity-based subject-independent pain assessment and highlight the potential of fNIRS for objective clinical pain monitoring.

## 1. Introduction

Pain is a pervasive public health problem that affects the quality of life, productivity, and healthcare systems worldwide [1,2,3]. Acute pain remains a leading cause of emergency presentations and postoperative complications, and inadequate assessment can lead to prolonged suffering and chronic pain [4,5,6,7,8]. The revised definition of pain emphasises its sensory and emotional components and highlights the subjective nature of the experience [9]. This subjectivity makes accurate clinical assessment difficult, yet reliable pain estimation is essential for diagnosis, treatment monitoring, and patient safety [10,11]. The challenge is particularly acute for nonverbal patients, individuals with impaired communication, and patients under sedation or anesthesia.

Current clinical practice relies heavily on self-report scales and clinician observation. Although these tools are widely used, they are limited by inter-rater variability, cultural and cognitive biases, and the inability to capture pain reliably when communication is compromised [8,10,11]. These limitations have driven research into objective pain assessment using physiological signals and behavioral cues [12,13,14,15,16]. Peripheral markers such as heart rate, respiration, and skin conductance have shown promise, including real-time wearable approaches, but they can be confounded by stress, medication, and comorbidities [12,13,17]. Facial-expression-based systems are sensitive to occlusion, conscious suppression, and motor impairments, and deep models still struggle to generalise across settings [14,15,16,18,19,20]. Recent systematic reviews of neurophysiological sensing for acute pain highlight both the promise and the need for robust subject-independent objective indicators of pain [21,22].

Neuroimaging provides a more direct avenue for objective pain assessment by capturing brain activity associated with nociceptive processing and pain perception [23,24]. Among available modalities, functional near-infrared spectroscopy (fNIRS) is particularly attractive due to its non-invasive nature, portability, tolerance to moderate motion, and favourable balance between temporal and spatial resolution, making it well suited to clinical and ecologically valid environments [25,26,27,28,29]. Although electroencephalography (EEG) offers higher temporal resolution, it is sensitive to electrical and muscular artefacts, susceptible to motion noise during nociceptive stimulation, and measures electrical surface potentials rather than the neurovascular responses that are directly relevant to cortical haemodynamic pain processing. In contrast, fNIRS directly reflects neurovascular coupling through changes in oxyhaemoglobin and deoxyhaemoglobin concentrations, is more tolerant of moderate body movement, and does not require conductive gel or complex electrode preparation—advantages that facilitate its deployment in clinical and experimental pain settings [25,26,27,29]. Furthermore, the prefrontal cortex, which is consistently implicated in the cognitive and affective modulation of pain, can be effectively covered by fNIRS optodes and produces robust haemodynamic responses to nociceptive stimulation [30,31], making fNIRS a particularly well-suited modality for the study of pain perception. Furthermore, its relatively low cost and practical deployment advantages facilitate data collection in settings where other neuroimaging techniques are less feasible. These characteristics support its growing potential for objective pain assessment and the identification of reliable neurophysiological indicators [23,24,27].

Machine learning approaches have significantly advanced fNIRS-based pain recognition by enabling the mapping of complex haemodynamic features to pain labels and improving generalisation beyond individual subjects [32,33,34,35,36,37,38]. Previous studies have explored a range of representations and modelling strategies, including functional data analysis, wavelet-based methods, statistical descriptors, and deep learning architectures [33,34,35,36,39]. Despite encouraging results, many studies rely on small cohorts or evaluation protocols that do not rigorously assess subject-independent performance. This limitation is critical, as pain responses exhibit substantial inter-individual variability, increasing the risk that models capture subject-specific characteristics rather than generalisable pain-related patterns. The importance of subject-independent evaluation is further emphasised by standardised pain assessment protocols and quantitative sensory testing frameworks, which highlight the variability in pain perception across individuals [40]. In addition, signal quality and preprocessing remain key challenges, as fNIRS measurements are susceptible to motion artefacts and physiological noise [41,42,43,44]. Collectively, these factors underscore the need for robust feature representations capable of generalising across subjects and recording conditions.

Functional and effective connectivity provide a network-level view of brain activity by quantifying interactions between regions rather than focusing on single-channel amplitudes [45]. Directed measures such as Granger causality can capture temporal precedence and directional influence, offering richer information than symmetric correlations [46]. Connectivity-based representations have been widely used in neuroscience to study network organisation and cognition, and methodological toolkits are now available to compute and analyse connectivity in a principled manner [45,47,48]. In EEG, connectivity features have recently been shown to improve classification in other domains such as mental workload, using hierarchical feature selection and machine learning [49], and have also been used to quantify pain severity from functional connectivity [50]. In fNIRS, connectivity analysis has been used to characterise functional networks and resting-state coupling [51,52], and effective connectivity has been applied to pain identification [53], while preprocessing strategies such as multiresolution and empirical mode decomposition (EMD)-based approaches address motion artifacts and physiological confounds [41,42,43,44,54,55,56]. However, despite these advances, connectivity-based features remain relatively underexplored for pain classification, particularly within subject-independent evaluation frameworks.

This paper addresses these gaps by proposing a connectivity-driven subject-independent framework for pain classification using fNIRS. The approach leverages functional and effective connectivity to capture network-level patterns associated with pain, enabling the modelling of inter-channel interactions rather than relying solely on channel-wise amplitudes. The framework is designed to provide a robust and interpretable pipeline that emphasises generalisation across subjects and supports objective pain assessment. Specifically, the contributions of this work are threefold: (1) the proposal of a connectivity-based feature representation for fNIRS pain classification that jointly captures functional and directed (effective) interactions between prefrontal channels—moving beyond the single-channel amplitude or statistical descriptors that dominate prior fNIRS pain decoding literature—and providing a richer network-level characterisation of pain-related cortical dynamics; (2) evaluation under a strict leave-one-subject-out protocol to ensure clinically meaningful subject-independent generalisation, together with a systematic mutual-information-based feature sweep that identifies the minimum feature count at which all three pain classes, including High Pain, are reliably detected; (3) an interpretable parsimonious pipeline that reduces the 1380-dimensional connectivity space to a 700-feature subset, demonstrating that a substantially smaller representation achieves performance comparable to the full feature set under strict cross-validation and providing a reproducible baseline that can be extended to future multimodal pain assessment studies.

## 2. Materials and Methods

### 2.1. Dataset and Protocol

**Figure 1:**
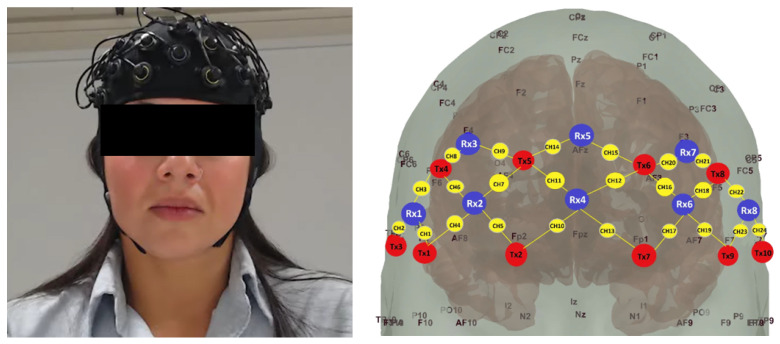
fNIRS channel layout. (**Left**): Participant wearing the fNIRS cap with optodes over the prefrontal region. (**Right**): Schematic of the 24 measurement channels (CH1–CH24) on a 3D head model with 10–20 system landmarks. Red circles indicate 10 sources (Tx1–Tx10), blue circles indicate 8 detectors (Rx1–Rx8), and yellow circles mark the midpoint of each source–detector pair forming the 24 channels. Channels are concentrated over the prefrontal and frontal cortex (Fp1, Fp2, Fz, AFz).

We used the AI4PAIN Grand Challenge dataset, which contains fNIRS recordings from 65 healthy participants (42 male, 23 female) collected over 24 prefrontal channels during controlled pain stimulation [57]. The participants’ age ranged from 17 to 52 years (mean 29.06, SD 8.28). Prior to the experiment, participants were screened to exclude chronic pain conditions, unstable medical issues, or recent medication use. Each participant was briefed about the study, and written informed consent was obtained before participation. The data collection was approved by the Human Research Ethics Committee of the University of Canberra (Ref. No. 11837). A wireless continuous-wave fNIRS system (Artinis Medical Systems, Gelderland, The Netherlands) recorded haemoglobin changes at 50 Hz using two wavelengths (760 and 840 nm). Optodes were arranged as 10 sources and 8 detectors with 35 mm source–detector spacing to cover the prefrontal cortex (Figure 1). The protocol includes baseline (No Pain), Low-Pain (LP), High-Pain (HP), and rest segments. Each stimulus lasts 10 s and is followed by a 40 s rest period. The dataset provides oxygenated (HbO_2_), deoxygenated (HHb), and total haemoglobin (HbT) signals.

### 2.2. Class Construction and Balance

Following data collection, the recorded signals were segmented into 10 s trials for each condition. Initial segmentation yielded, per participant, 6 trials for the No Pain (NP) condition (recorded prior to stimulus onset), 12 trials for Low Pain (LP), and 12 trials for High Pain (HP), resulting in an initial class distribution of 6:12:12. To mitigate the class imbalance, an additional 6 NP trials were extracted from rest periods preceding subsequent pain stimulations. Consequently, the NP condition included recordings obtained both before and after stimulation, enabling a more representative characterisation of the No-Pain state and capturing physiological recovery dynamics. After balancing, each participant contributed 12 trials per class, yielding 36 trials per subject. The final dataset comprised 2340 observations in total (780 per class), collected from 65 participants, each contributing 12 samples per pain condition.

### 2.3. Preprocessing

Raw fNIRS data are provided as session-separated files with 24 channels of oxygenated (HbO_2_) and 24 of deoxygenated (HHb) haemoglobin, interleaved in columns, sampled at 50 Hz. The preprocessing pipeline was applied in the following order. First, noisy channels were detected and corrected. For each channel, the coefficient of variation (CV) was computed as the ratio of standard deviation to mean absolute signal over the session. Channels with CV above the 95th percentile across all channels were flagged as faulty. Channels 19 and 21 were consistently identified across sessions and corrected by spatial interpolation: at each time point, channel 19 was replaced by the mean of channels 17, 18, and 23 and channel 21 by the mean of channels 20, 18, and 22, so that all 24 channels were retained. This interpolation strategy affects only 2 of 24 channels (8.3% of the network). Because the replacement values are derived from the immediate spatial neighbours of the faulty channels—channels that are physically adjacent and therefore expected to share similar haemodynamic signals—the interpolated time series are strongly correlated with their neighbourhood by construction. This does introduce a degree of artificial spatial correlation between the interpolated channels and their neighbours; however, the effect is local and involves a small fraction of the total connectivity matrix. The interpolation also ensures that the full 24-channel network topology is preserved, avoiding the distortion that would arise from dropping channels and reindexing the connectivity matrices. We acknowledge this as a methodological limitation and recommend that future studies use hardware-level channel replacement or short-channel regression to avoid interpolation artefacts altogether. Second, the same five-step pipeline was applied separately to HbO_2_ and HHb. Step 1: Baseline correction—per-channel mean subtraction to center each time series and remove session-level amplitude offsets that vary across participants and recording sessions. Step 2: Artifact detection and local calibration—segments exceeding three standard deviations from the channel mean were identified, and a 6 s windowed mean correction was applied to reduce motion-related offsets; this threshold and window length were selected to be sensitive to sharp transient motion spikes while avoiding the removal of genuine haemodynamic responses, which evolve more slowly. Step 3: Band-pass filtering—a fourth-order Butterworth filter (0.01–0.2 Hz) with zero-phase forward–backward implementation to retain task-related haemodynamic fluctuations and attenuate slow drifts and high-frequency noise; the lower cutoff of 0.01 Hz removes very low-frequency baseline drift, and the upper cutoff of 0.2 Hz is above the canonical haemodynamic response frequency band (0.01–0.1 Hz) while still suppressing cardiac and respiratory artefacts, in line with standard fNIRS preprocessing practice [27,41]. Step 4: Wavelet denoising—Daubechies-4 wavelets with soft thresholding to reduce residual high-frequency artifacts; Daubechies-4 wavelets were chosen for their compact support and near-linear phase properties, which preserve signal morphology while effectively suppressing noise that survives band-pass filtering [42]. Step 5: Feature scaling—per-channel min–max scaling to [0, 1] for consistent input scaling across channels; this step ensures that channels with larger absolute amplitude do not dominate the correlation and partial-correlation connectivity measures, and it is applied per-channel so that relative temporal dynamics are preserved. Third, total haemoglobin (HbT) was computed as HbT = HbO_2_ + HHb after preprocessing.

### 2.4. Connectivity Feature Extraction

For each 10 s trial and each haemoglobin type (HbO_2_, HHb, HbT), we compute the functional connectivity (Pearson correlation, partial correlation, coherence) and effective connectivity (Granger causality). For channels *i* and *j*, the Pearson correlation is

(1) $$r_{ij}=\frac{\mathrm{cov}(x_{i},x_{j})}{\sigma_{i}\sigma_{j}}$$

where $x_{i}$ and $x_{j}$ are the trial time series, and σ is the standard deviation. Partial correlation is obtained from the precision matrix $P=\Sigma^{-1}$ as

(2) $$\rho_{ij}=-\frac{P_{ij}}{\sqrt{P_{ii}P_{jj}}}$$

which measures direct coupling while controlling for other channels. Coherence captures the frequency-domain coupling and is defined as

(3) $$C_{ij}\left(f\right)=\frac{|S_{ij}\left(f\right){|}^{2}}{S_{ii}\left(f\right)S_{jj}\left(f\right)}$$

where $S_{ij}\left(f\right)$ is the cross-spectral density. Granger causality models the directional influence using a multivariate autoregressive formulation; channel *j* is said to Granger-cause *i* if including past values of $x_{j}$ significantly improves the prediction of $x_{i}$ [46]. We vectorise the upper triangle (excluding the diagonal) for correlation, partial correlation, and coherence, yielding 24×23/2 features per matrix, and use all off-diagonal entries for Granger causality to retain directionality. Concatenating these components yields a 1380-dimensional connectivity feature vector per trial, capturing network-level interactions rather than individual channel amplitudes.

### 2.5. Feature Selection

To reduce dimensionality and improve interpretability, we apply mutual-information-based feature selection within each training fold. Features are ranked by their mutual information with the class label, and the top *k* features are retained for training the classifier. We chose mutual information because it captures non-linear dependencies between features and class labels, which suits tree-based and kernel classifiers better than univariate methods such as the ANOVA F-test [58,59,60]. Mutual information does not assume linearity or normality, making it well suited for haemodynamic connectivity features that may exhibit complex non-linear relationships with pain states.

The number of features *k* is treated as a hyperparameter. We conduct a systematic sweep across a range of candidate values—from 1 to 1380—to evaluate the classification performance as a function of the feature count. For each value of *k*, the top *k* MI-ranked features are selected within each LOSO training fold, and the classifier is trained and evaluated on the held-out subject. This procedure is repeated for each haemodynamic modality (HbO_2_, HHb, HbT) and for each classifier (SVM, KNN, RF, XGBoost). The sweep is performed independently per modality, so that feature rankings and selected subsets may differ across HbO_2_, HHb, and HbT.

### 2.6. Classification and Evaluation

We evaluate multiple well-known classifiers: SVM, KNN, RF, and XGBoost. To avoid overfitting in this limited-sample setting and to keep the pipeline reproducible, we train all classifiers with their default hyperparameters and do not perform hyperparameter tuning. The overall evaluation uses leave-one-subject-out (LOSO) cross-validation: each subject is held out once for testing, and the performance metrics are averaged across subjects. It should be noted that using default hyperparameters, while improving reproducibility, may not yield the optimal performance for each individual classifier and may limit the fairness of cross-classifier comparisons. Future work should include systematic hyperparameter optimisation, for example via nested cross-validation, to provide a more equitable performance benchmark.

### 2.7. Evaluation Metrics

The performance is reported using accuracy, precision, recall, and F1-score. Let TP, TN, FP, and FN denote true positives, true negatives, false positives, and false negatives, respectively. Then,

(4) $$\begin{array}{cc} \mathrm{Accuracy} & =\frac{TP+TN}{TP+TN+FP+FN} \end{array}$$

(5) $$\begin{array}{cc} \mathrm{Precision} & =\frac{TP}{TP+FP} \end{array}$$

(6) $$\begin{array}{cc} \mathrm{Recall} & =\frac{TP}{TP+FN} \end{array}$$

(7) $$\begin{array}{cc} F1 & =\frac{2TP}{2TP+FP+FN} \end{array}$$

### 2.8. Framework Overview

**Figure 2:**
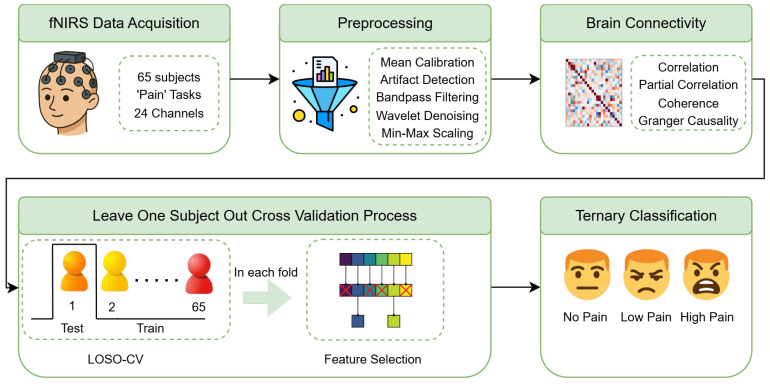
Overview of the proposed methodology: fNIRS data acquisition, preprocessing, functional and effective connectivity computation, mutual-information-based feature selection, ternary classification (NP vs. LP vs. HP), and LOSO evaluation.

Figure 2 provides a high-level view of the proposed pipeline, from data acquisition and preprocessing to connectivity computation, mutual-information-based feature selection, ternary classification, and evaluation under LOSO cross-validation.

## 3. Results

This section presents the experimental results obtained from the proposed connectivity-based framework for pain recognition using fNIRS signals. First, the connectivity matrices derived from the prefrontal cortex channels are examined to characterise the functional interactions between cortical regions. Next, we report the classification performance using the full set of extracted connectivity features, evaluating multiple machine learning models under a subject-independent evaluation protocol. We then present the classification results after feature selection, highlighting the performance achieved with a reduced set of informative features and assessing the benefits of a more compact representation. Finally, we analyse the frequently selected connectivity features that consistently contribute to pain discrimination across cross-validation folds. This analysis provides insight into the most informative connectivity patterns for subject-independent pain recognition.

### 3.1. Connectivity Matrices

**Figure 3:**
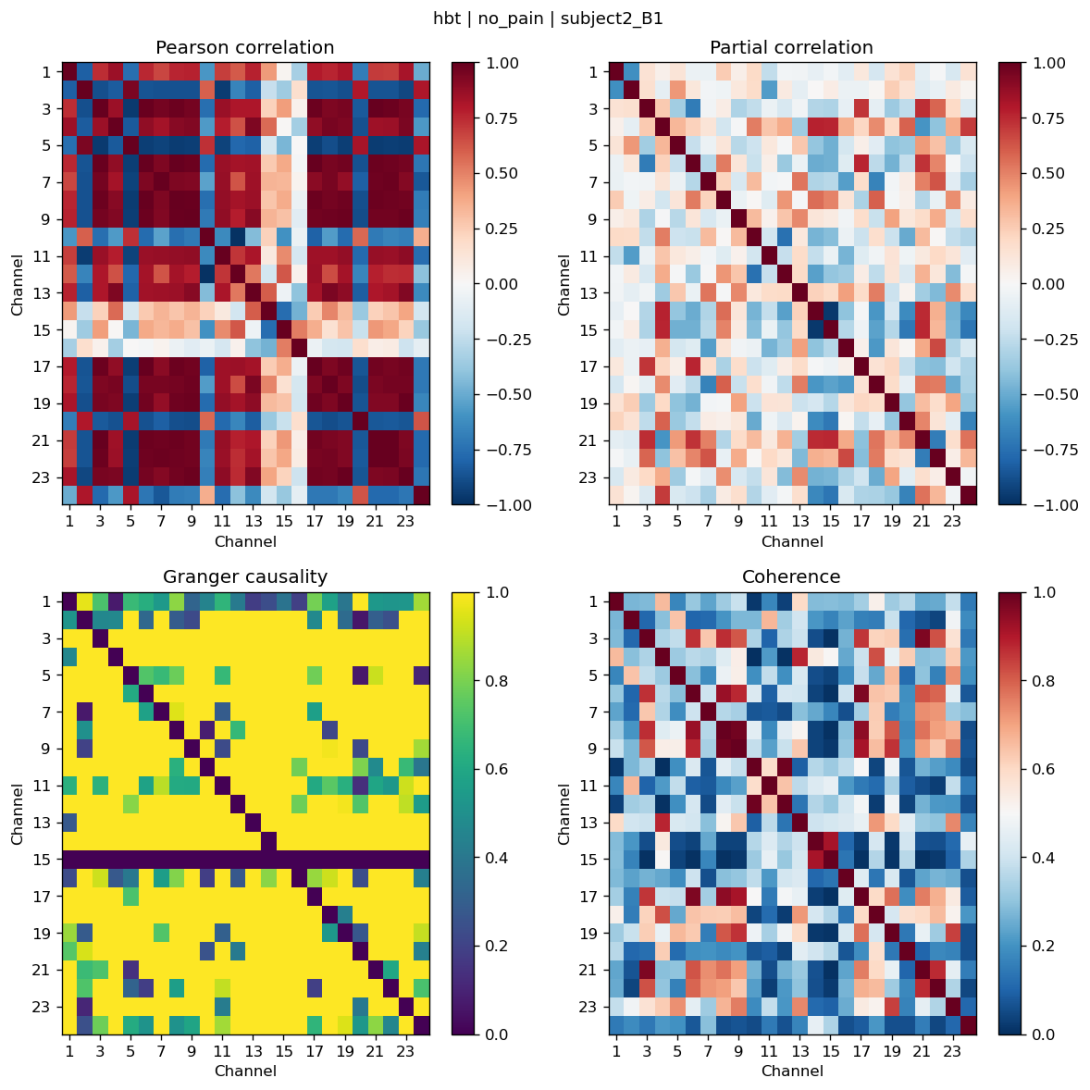
Sample connectivity matrices for HbT in the No-Pain condition (subject 2, session B1). From (**left**) to (**right**): Pearson correlation, partial correlation, Granger causality, and coherence (all 24 × 24).

Figure 3 shows an example of the four connectivity matrices (Pearson correlation, partial correlation, Granger causality, and coherence) computed for a single HbT trial in the No-Pain condition (subject 2, session B1). Each matrix is 24 × 24, corresponding to the 24 prefrontal fNIRS channels. The correlation matrix exhibits a symmetric structure with moderate to strong positive values along and near the diagonal, reflecting coherent haemodynamic fluctuations between neighbouring channels. The partial correlation matrix, which removes indirect effects, shows a sparser pattern with both positive and negative entries, highlighting direct pairwise associations. The Granger causality matrix is asymmetric and captures directed influences between channels; off-diagonal entries indicate temporal precedence, with stronger values suggesting more consistent directional coupling. The coherence matrix reflects frequency-dependent synchronisation, with elevated values in specific channel pairs indicating shared oscillatory activity. Across the four measures, certain channel pairs show consistently stronger connectivity than others, suggesting regions of the prefrontal network with more pronounced inter-channel coupling. The structure of these matrices forms the basis of the 1380-dimensional connectivity feature vector used for classification.

### 3.2. Results Using All Features

Table 1 reports the ternary classification performance (NP vs. LP vs. HP) under LOSO cross-validation when using all 1380 connectivity features for each haemodynamic modality. Overall, all modalities achieved comparable performance, with classification accuracies ranging from approximately 65% to 70%, depending on the model and haemodynamic signal. Among the evaluated models, Random Forest (RF) achieved the best performance for the HbO_2_ modality, reaching an accuracy of 69.1%, with corresponding precision, recall, and F1-scores of 69.3%, 69.1%, and 68.4%, respectively. For the HHb modality, the SVM classifier achieved the highest accuracy of 69.1%, with consistent precision, recall, and F1-scores across folds. The best overall performance was observed for the HbT modality, where SVM achieved the highest accuracy of 69.9%, along with the strongest precision (70.4%) and F1-score (69.2%). Across modalities, KNN consistently showed lower performance, with accuracies between 65.7% and 67.0%, while XGBoost and RF provided competitive results close to the best-performing models. These results indicate that the connectivity features derived from fNIRS signals contain discriminative information for subject-independent pain recognition, although the use of the full high-dimensional feature set does not necessarily provide substantial performance gains across all models.

### 3.3. Results After Feature Selection

**Figure 4:**
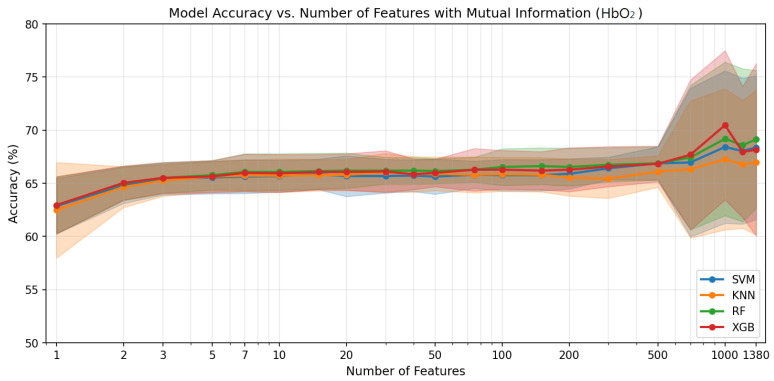
Accuracy vs. number of features for HbO_2_. Mean accuracy (solid lines) and standard deviation across LOSO folds (shaded regions) for each classifier.

**Figure 5:**
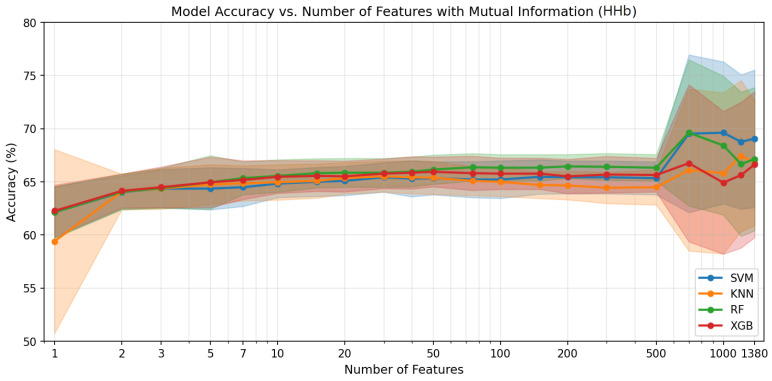
Accuracy vs. number of features for HHb. Mean accuracy (solid lines) and standard deviation across LOSO folds (shaded regions) for each classifier.

**Figure 6:**
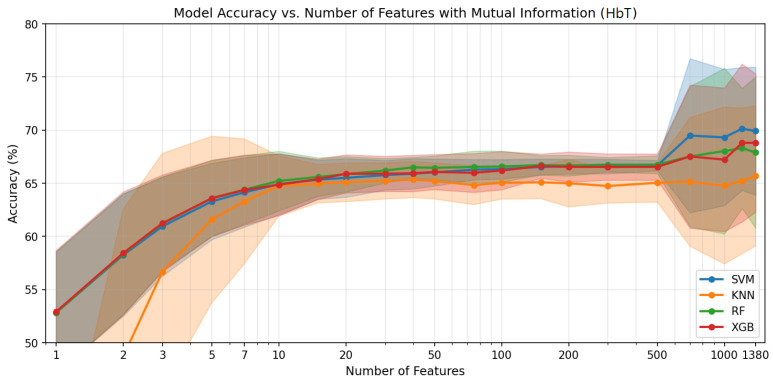
Accuracy vs. number of features for HbT. Mean accuracy (solid lines) and standard deviation across LOSO folds (shaded regions) for each classifier.

Figure 4, Figure 5 and Figure 6 show the classification accuracy versus the number of selected features for each haemodynamic modality across the four classifiers. The performance generally improves with the increasing feature count up to several hundred features, after which the gains diminish and the variability increases. Table 2 summarises the ternary classification results obtained after applying feature selection. Overall, feature selection slightly improved performance across modalities while reducing the dimensionality of the connectivity feature space. For the HbO_2_ modality, the best performance was achieved by XGBoost, reaching an accuracy of 70.5% using 1000 selected features. For HHb, the highest accuracy (69.6%) was obtained with SVM, with Random Forest achieving comparable performance using only 700 features. For HbT, SVM achieved the best result with an accuracy of 70.1% using 1200 features. Across modalities, SVM, RF, and XGBoost produced competitive results, while KNN consistently showed lower performance, indicating that informative connectivity patterns can be captured with a reduced set of features without compromising classification performance.

### 3.4. Results for the Proposed Model

The feature selection sweep revealed a clear transition in classification behaviour as the number of selected features increased. For n≤500, the mean recall for the High Pain (HP) class remains extremely low (∼0.7–4.2%), indicating that the models largely fail to identify HP instances and effectively discriminate only between the No Pain (NP) and Low Pain (LP) classes. A substantial improvement in HP detection is observed only when n≥700, where HP recall increases sharply to approximately 50–51%.

Table 3 summarises the classification performance in terms of accuracy, macro F1-score, and HP recall (mean ± standard deviation across modalities) for different feature subset sizes. Increasing the number of features beyond n=700 provides only marginal improvements in performance. For example, the accuracy increases slightly from 67.5% at n=700 to 67.8% at n=1000, while the macro F1 increases from 67.1% to 67.3%, and HP recall improves marginally from 50.0% to 50.6%. These small gains suggest a performance plateau, where additional features provide limited discriminative information.

To choose among candidate feature counts in a principled way, we defined the following selection criterion *a priori*: the proposed configuration is the smallest *n* at which (i) HP recall first reaches and stabilises at or above 50%—the point at which all three classes are detected at a level clearly above chance for a three-class task—and (ii) overall accuracy and macro F1 reach within 1 percentage point of the values obtained with the full 1380-feature model. HP recall was chosen as the primary constraint because, for n≤500, the classifier effectively collapses to a two-class problem by almost never predicting HP (see Table 3); thus, HP detectability serves as a qualitative breakpoint rather than a continuous optimisation target. The 50% threshold is not presented as a clinically sufficient detection rate but rather as the minimum requirement for the model to exhibit any meaningful ternary discrimination. By this criterion, n=700 is the smallest configuration that satisfies both requirements, and we adopt it as the proposed model.

Considering this trade-off between predictive performance and model complexity, we select n=700 as the proposed feature subset. It is important to note that the reported HP recall of approximately 50% means that, on average, half of all High Pain instances are correctly identified. This is a meaningful improvement over the near-zero HP recall at n≤500, but it also means that the remaining half of HP instances are misclassified—a limitation that is discussed in Section 4. This configuration achieves competitive overall performance (accuracy∼67.5% and macro F1∼67.1%) using approximately half of the full feature set (700 vs. 1380 features), while supporting a more compact and interpretable model.

Using 700 connectivity features selected via mutual information ranking, the proposed model achieved competitive performance across modalities while substantially reducing the feature dimensionality. For HbO_2_, XGBoost achieved an accuracy of 67.7±7.1% with an F1-score of 67.1±7.2%. For HHb, Random Forest achieved the best performance, reaching 69.6±6.9% accuracy and 69.4±6.9% F1-score. For HbT, SVM achieved 69.5±7.3% accuracy and 68.8±7.6% F1-score. Notably, the HHb modality consistently provided the strongest and most stable classification performance with a reduced feature set, suggesting that HHb-based connectivity patterns may represent a promising candidate biomarker for subject-independent pain recognition. Importantly, the selected subset of 700 features corresponds to approximately half of the full feature set (1380 features), yet the obtained results remain comparable to the best configurations that use 1000–1380 features (Table 2). This demonstrates that a substantially smaller set of informative connectivity features can maintain reliable detection of High-Pain (HP) states while supporting a more simplified model.

#### Per-Class Performance of the Proposed 700-Feature Model

To provide a more transparent view of the ternary classification task, Table 4 reports per-class recall (mean ± standard deviation across LOSO folds) for each modality and best classifier at n=700 features, computed directly from the fold-level results. This breakdown makes explicit the asymmetry in the per-class performance: the model achieves substantially higher recall for the NP class (∼98–100%) than for the LP (∼53–58%) and HP (∼50–55%) classes, confirming that HP detection remains the primary challenge in ternary pain classification. The large within-class standard deviations (13–18% for LP and HP) further indicate substantial variability in per-class detection across subjects.

### 3.5. Frequently Selected Connectivity Features

For interpretability, we performed an exploratory analysis of the most stable connectivity features. Table 5 lists the five features that appeared most frequently among the strongest-ranked (top 10) mutual-information features across the 65 LOSO folds. This analysis is exploratory and serves interpretability only; the proposed classification model uses 700 features. The count indicates the number of folds in which each feature was in the top 10 (maximum 65 per modality); the percentage is the proportion of folds (count/65).

Across modalities, several connections appear consistently within the top-ranked features. In particular, for the HbO_2_ modality, the connection granger_ch05_to_ch20 was selected in 63 out of 65 folds (96.9%), indicating a highly stable connectivity feature. Similarly, HHb and HbT modalities show repeated selection of specific channel pairs, suggesting that certain directed interactions between prefrontal channels are consistently informative for pain recognition. These findings reinforce the compact nature of the proposed framework, demonstrating that a small subset of stable connectivity features can capture meaningful neural signatures associated with pain.

## 4. Discussion

This study demonstrates that functional and effective connectivity features derived from fNIRS can yield meaningful subject-independent ternary pain classification (NP vs. LP vs. HP). The obtained accuracies indicate that connectivity patterns between prefrontal channels carry pain-related information that generalises across individuals. Unlike time-domain features, which summarise localised signal statistics, connectivity features describe coordinated network activity and directional influence, providing a higher-level representation of pain-related neural dynamics [27,45,46]. Similar connectivity-based feature pipelines have been effective in other classification domains such as mental workload and pain severity quantification, supporting the general value of connectivity for discriminative modelling [49,50]. This network perspective aligns with the distributed nature of pain processing, where multiple cortical regions interact to encode intensity and affective components of pain [23,24,61].

Across evaluated classifiers and modalities, SVM, Random Forest, and XGBoost produced competitive results, with the best model varying by modality. For the proposed 700-feature model, XGBoost performed best for HbO_2_, Random Forest for HHb, and SVM for HbT. Accuracies in the range of 67.7–69.6% compare favourably with prior fNIRS pain studies that rely on time-domain features or smaller cohorts [33,34,35,36,37,38] and with recent AI4PAIN baseline and multimodal benchmarks, where overall multiclass accuracies remain modest [19,20,57]. The comparable performance of HHb indicates that deoxygenation signals, despite lower signal-to-noise ratio, can be informative when encoded as connectivity [62]. For the proposed model, HHb achieves the highest ternary accuracy (69.6%), followed by HbT (69.5%) and HbO_2_ (67.7%), suggesting that HHb and HbT connectivity patterns carry a slight advantage when separating three pain levels [27,36,37].

Importantly, the feature selection sweep showed that comparable performance can be achieved with a substantially reduced feature set. As shown in Table 1 and Table 3, using all 1380 connectivity features yields mean accuracy up to 69.9% but with large standard deviations (6–8%) across LOSO folds. For n≤500, High Pain recall remains very low (∼0.7–4.2%), whereas with n=700 features, HP recall reaches ∼50%, and accuracy reaches 69.6% (HHb, Random Forest). We chose n=700 as the proposed configuration: it achieves performance comparable to the best large-feature models while using approximately half the features (700 vs. 1380) and constituting the minimum configuration at which ternary classification is viable. The HP recall of ∼50% represents a substantial improvement over the near-zero HP recall at n≤500; however, it should be framed carefully. A 50% recall means that one in two High Pain instances goes undetected, which is a meaningful limitation in any application where HP identification is clinically important. This result should be read as evidence that connectivity features contain signals relevant to HP discrimination, but not as evidence that the model is ready for clinical pain monitoring. Emphasising a reduced feature set offers several advantages: it improves interpretability, reduces computational complexity, mitigates overfitting risk, and enhances generalisability, particularly in limited-sample datasets. The low standard deviation across LOSO folds indicates stable and robust performance, suggesting that the model generalises well across subjects rather than relying on subject-specific patterns. Framing the approach around efficiency and robustness—rather than maximising the feature count—supports the use of compact connectivity biomarkers for pain assessment as a proof-of-concept framework.

The ternary classification task is inherently more challenging, as it requires discriminating between two adjacent pain intensity levels. The observed accuracies indicate that meaningful separation is achieved; however, the performance remains constrained, likely due to the partial overlap between Low- and High-Pain states. This trend is consistent with prior studies reporting reduced discriminative performance when adjacent pain intensities must be distinguished [37,57]. These findings suggest that the neurophysiological signatures of LP and HP exhibit overlapping connectivity patterns within the prefrontal cortex. Incorporating complementary modalities—such as peripheral physiological signals, facial behaviour, or multimodal fusion strategies—may enhance fine-grained discrimination and improve the overall classification performance [12,14,19,20].

Table 6 compares the proposed approach with prior fNIRS-based pain classification studies. Earlier work on smaller cohorts (*N* = 18–43) reports high accuracies for binary (80–94%) and multiclass (90–94%) tasks using temporal, frequency, or wavelet features with SVM or deep learning [33,34,35,36,38,63], but limited sample sizes may inflate performance. On the larger AI4PAIN dataset (*N* = 65), the baseline SVM achieved 43.20% multiclass accuracy [57], while an ensemble using engineered temporal features reached 68.20% multiclass and 91.41% binary [64]. The proposed connectivity-driven framework achieves 69.6% ternary accuracy (HHb, Random Forest, 700 features) under strict LOSO validation with a reduced feature set, placing it competitively among subject-independent methods on this dataset. Unlike prior work focused on single-channel amplitude or statistical features, the proposed method models inter-channel interactions via functional and effective connectivity, offering a richer and potentially more physiologically meaningful representation at the cost of higher complexity under subject-independent evaluation. Direct performance comparisons across these studies should be interpreted with caution. Differences in cohort size, pain stimulation protocol, class definition, validation strategy, and feature extraction approach make numerical comparisons approximate. In particular, studies using within-subject or leave-one-trial-out cross-validation may report substantially higher accuracies than LOSO protocols, as the latter must generalise across individuals rather than within a single subject’s recording. The comparison is therefore primarily intended to situate the proposed work in the literature rather than to make claims of superiority. These results underscore the value of large-cohort validation and connectivity-based modelling for pain assessment research.

The exploratory frequent-feature analysis provides interpretable insight into which connections are most informative among the strongest-ranked features. Based on Table 5, the most stable features are predominantly Granger-causality connections, indicating that features encoding directed temporal influence between channels are especially discriminative for pain recognition [46]. As a post hoc sensitivity check for the interpolation of channels 19 and 21 (Section 2.3), we note that Granger-causality edges involving these channels still appeared among the most frequently selected features across folds (e.g., granger_ch18_to_ch21 for HbO_2_, granger_ch06_to_ch19 and granger_ch05_to_ch19 for HHb, granger_ch17_to_ch21 and granger_ch19_to_ch08 for HbT; see Table 5), suggesting that these channels retained discriminative information relevant to pain classification despite the spatial interpolation. This is consistent with the interpretation that the local artificial correlation introduced by interpolation did not eliminate the pain-relevant temporal structure in those channels. This finding should be interpreted carefully. Granger causality in fNIRS reflects predictive temporal precedence in haemodynamic signals: a Granger-causality edge from channel *j* to channel *i* means that past values of the HbO_2_/HHb signal at *j* improve the autoregressive prediction of *i*. Given the haemodynamic nature of fNIRS signals, the relatively short 10 s analysis windows, and the known sensitivity of Granger-based measures to preprocessing and noise, we do not claim that these features reflect genuine directed neural information flow or causal connectivity in a neurophysiological sense [45,46]. Rather, the data support that Granger-causality features are more predictively useful for pain class discrimination than symmetric functional connectivity measures, suggesting that temporal asymmetry in haemodynamic signals carries class-relevant information beyond that captured by symmetric correlations. In HbO_2_, the most frequently selected feature (granger_ch05_to_ch20) appeared in 63 of 65 folds, with other recurring edges involving channels 14, 18, 20, and 21. In HHb, directed edges toward channel 19 (e.g., granger_ch06_to_ch19, granger_ch05_to_ch19) recur, alongside channels 2, 3, 5, and 17. In HbT, granger_ch17_to_ch21 and granger_ch07_to_ch10 are prominent, with channels 8, 10, 14, and 23 also appearing. Since the recordings are limited to prefrontal channels, these patterns should not be interpreted as evidence of broader pain-network dynamics. Rather, they reflect haemodynamic interactions within the prefrontal cortex, a region consistently involved in the cognitive and affective modulation of pain [30,31]. While channel indices do not directly map to single anatomical areas without optode localisation, they likely correspond to neighbouring prefrontal subregions. The repeated selection of these edges implies that pain modulates haemodynamic temporal structure in a structured way rather than producing diffuse global changes. Feature frequency acts as a proxy for reliability: edges that are repeatedly selected across subjects are less likely to be idiosyncratic and more likely to reflect common pain-related dynamics. This aligns with the broader goal of identifying neural biomarkers that are robust across individuals [23,24,27].

In addition, the exploratory analysis of the most frequently selected connectivity features (among the top 10 ranked) provides insight into which connections are most stable for interpretability. Although the proposed model uses 700 features, connectivity features that consistently appear among the strongest-ranked candidates across LOSO folds are less likely to reflect fold-specific variability and more likely to represent stable functional interactions between cortical regions. Such stability is particularly important in subject-independent models, where feature robustness across individuals is critical for generalisation. However, it is important to note that this stability analysis covers only the top 10 features per fold and does not establish the reproducibility of the full 700-feature subset used for classification. Feature selection is performed independently within each training fold; so, the composition of the 700-feature set may vary across folds. While the dominance of Granger-causality edges in the top-10 analysis suggests a consistent pattern, the broader 700-feature representation may include fold-specific features that contribute to within-fold performance but may not generalise robustly. Future work should employ dedicated feature-stability metrics—such as Jaccard overlap between fold-specific feature sets or rank-based stability indices—applied to the full 700-feature subset to more rigorously assess the reproducibility of the proposed representation. Previous neuroimaging studies have shown that pain perception involves coordinated activity across distributed cortical networks rather than isolated regional responses [65,66]. In particular, the prefrontal cortex has been repeatedly implicated in the cognitive and affective modulation of pain and has demonstrated measurable haemodynamic responses in fNIRS-based pain studies [67,68]. The repeated selection of specific directed connectivity links in our results suggests that pain-related neural dynamics may be characterised by a limited set of stable interactions within these cortical networks. Similar findings have been reported in functional connectivity analyses of pain processing using both fMRI and fNIRS, where consistent connectivity patterns were associated with nociceptive processing and pain modulation [27,52,69]. The presence of such recurrent connections supports the hypothesis that pain perception is mediated by stable network-level interactions and suggests that these connectivity patterns may represent potential biomarkers for objective pain assessment. Importantly, identifying a small subset of reproducible connectivity features aligns with the modelling approach adopted in this work, reinforcing the feasibility of developing interpretable and generalisable pain recognition systems based on a reduced number of informative neural connections.

The results are encouraging, but several limitations should be noted. Most critically, the HP recall of ≈50% means that half of all High Pain instances are missed. The asymmetric clinical cost of failing to detect a true High Pain state substantially outweighs that of misclassifying a No Pain instance; the current framework does not account for this risk, and cost-sensitive learning should be explored in future work. The study also uses healthy participants under controlled electrical stimulation, constituting a proof-of-concept that must be validated in clinical cohorts before any claims of clinical readiness can be made [21,22]. LOSO provides strict subject-independent evaluation, yet it does not reflect real-world clinical complexity. Connectivity features were derived from fixed 10 s windows, assuming stationarity despite the dynamic nature of pain processing; temporal or dynamic connectivity methods may better capture evolving responses. Granger-causality features, while predictively useful, are sensitive to preprocessing and short analysis windows, and their robustness to alternative pipelines warrants future investigation [41,42,43,44,46]. Longitudinal studies are needed to assess whether these connectivity patterns are stable across sessions.

Despite these limitations, the present study introduces a practical and interpretable connectivity-based framework for objective pain assessment. Mutual-information-based feature selection with a systematic sweep yields compact reproducible feature subsets and supports transparent analysis of connectivity patterns associated with pain. This reduced-feature approach improves interpretability, lowers computational cost, mitigates overfitting, and enhances generalisability in limited-sample settings. The repeated selection of specific directed connections indicates that effective connectivity in the prefrontal cortex encodes pain-related dynamics in a stable, subject-independent manner. These findings extend prior fNIRS pain decoding work by emphasising network-level representations over single-channel amplitude features. The pipeline balances performance and interpretability, can be run with modest computational resources, and produces a substantially reduced set of physiologically meaningful connectivity features that can be examined and compared across studies. Such transparency is important for clinical adoption, where robustness, reproducibility, and explainability matter, and clinicians may prefer a concise set of interpretable indicators over opaque high-dimensional models.

## 5. Conclusions

We presented a subject-independent framework for ternary pain classification (NP vs. LP vs. HP) using fNIRS functional and effective connectivity features. On the AI4PAIN dataset—collected from healthy participants under controlled experimental pain—we identified a 700-feature connectivity subset that achieves competitive classification performance (up to 69.6% accuracy for HHb) under strict LOSO validation, with performance comparable to models using the full 1380 features, and constituting the minimum configuration at which High Pain instances begin to be detected (HP recall ≈ 50%). It should be emphasised that an HP recall of 50% reflects a proof-of-concept result: the model identifies High Pain at a rate meaningfully above chance for a three-class problem but misses the remaining half of HP instances—a limitation that must be addressed before any clinical deployment. The current results are not intended to imply clinical readiness, but rather to demonstrate the feasibility of connectivity-based subject-independent pain decoding and to provide a reproducible baseline for future improvements. Notably, Granger-causality features dominated the frequently selected top-ranked features across all haemodynamic modalities, suggesting that temporal asymmetry in prefrontal haemodynamic signals provides predictive information beyond symmetric functional connectivity; however, this should not be interpreted as direct evidence of causal neural information flow given the haemodynamic and short-window nature of the data. Emphasising model parsimony yields interpretable, computationally efficient, and generalisable models suited to limited-sample experimental settings. Future work should address the low HP recall through cost-sensitive learning, validate the framework in clinical patient cohorts, and employ formal feature-stability assessment for the full 700-feature representation. These findings provide a reproducible baseline that can be extended to future multimodal and clinical pain assessment studies.

## Figures and Tables

**Table 1:** Ternary classification results with all 1380 connectivity features (NP vs. LP vs. HP, LOSO). Mean ± standard deviation across folds (%). Best accuracy per modality in bold.

Modality	Model	Accuracy	Precision	Recall	F1
HbO_2_	SVM	68.4 ± 6.8	68.6 ± 7.5	68.4 ± 6.8	67.7 ± 7.0
	KNN	67.0 ± 6.8	66.9 ± 7.2	67.0 ± 6.8	66.5 ± 6.9
	RF	**69.1 ± 6.5**	**69.3 ± 7.1**	**69.1 ± 6.5**	**68.4 ± 6.9**
	XGBoost	68.2 ± 8.1	68.3 ± 9.4	68.2 ± 8.1	67.2 ± 8.6
HHb	SVM	**69.1 ± 6.5**	**69.2 ± 7.1**	**69.1 ± 6.5**	**68.5 ± 6.8**
	KNN	66.8 ± 5.9	65.9 ± 6.7	66.8 ± 5.9	65.8 ± 6.3
	RF	67.1 ± 6.7	67.5 ± 6.9	67.1 ± 6.7	66.9 ± 6.8
	XGBoost	66.6 ± 6.9	66.8 ± 7.3	66.6 ± 6.9	66.3 ± 6.9
HbT	SVM	**69.9 ± 6.0**	**70.4 ± 6.7**	**69.9 ± 6.0**	**69.2 ± 6.1**
	KNN	65.7 ± 6.6	65.9 ± 6.7	65.7 ± 6.6	65.3 ± 6.6
	RF	67.9 ± 7.1	68.2 ± 7.6	67.9 ± 7.1	67.4 ± 7.1
	XGBoost	68.8 ± 6.5	69.0 ± 6.9	68.8 ± 6.5	68.4 ± 6.6

**Table 2:** Best ternary classification results for each classifier and modality after feature selection (NP vs. LP vs. HP, LOSO). Mean ± standard deviation across folds (%). Best accuracy per modality in bold.

Modality	Model	N Features	Accuracy	Precision	Recall	F1
HbO_2_	SVM	1000	68.4 ± 7.2	68.5 ± 7.7	68.4 ± 7.2	67.8 ± 7.4
	KNN	1000	67.3 ± 6.6	67.3 ± 6.9	67.3 ± 6.6	66.9 ± 6.7
	RF	1000	69.2 ± 7.2	69.2 ± 7.6	69.2 ± 7.2	68.6 ± 7.5
	XGBoost	1000	**70.5 ± 7.0**	**70.8 ± 7.7**	**70.5 ± 7.0**	**69.9 ± 7.2**
HHb	SVM	1000	**69.6 ± 6.7**	**69.9 ± 7.2**	**69.6 ± 6.7**	**69.0 ± 6.9**
	KNN	1200	67.4 ± 7.1	67.7 ± 7.7	67.4 ± 7.1	67.1 ± 7.1
	RF	700	**69.6 ± 6.9**	**70.1 ± 6.9**	**69.6 ± 6.9**	**69.4 ± 6.9**
	XGBoost	700	66.8 ± 7.4	67.2 ± 7.5	66.8 ± 7.4	66.5 ± 7.4
HbT	SVM	1200	**70.1 ± 5.8**	**70.4 ± 6.4**	**70.1 ± 5.8**	**69.4 ± 6.1**
	KNN	1380	65.7 ± 6.6	65.9 ± 6.7	65.7 ± 6.6	65.3 ± 6.6
	RF	1200	68.3 ± 5.6	68.4 ± 6.2	68.3 ± 5.6	67.7 ± 5.8
	XGBoost	1200	68.8 ± 7.4	69.0 ± 8.1	68.8 ± 7.4	68.1 ± 7.7

**Table 3:** Summary of classification performance versus number of features. Values are the mean across the three modalities (HbO_2_, HHb, HbT) for each feature count, with standard deviation across modalities.

N Features	Accuracy (%)	F1 (Macro) (%)	Recall HP (%)
10	65.3 ± 0.4	54.7 ± 0.5	4.2 ± 3.5
20	65.7 ± 0.3	55.0 ± 0.4	1.3 ± 1.0
50	65.9 ± 0.4	55.2 ± 0.4	0.7 ± 0.4
100	65.9 ± 0.5	55.3 ± 0.5	1.1 ± 0.6
300	66.0 ± 0.8	55.4 ± 0.7	1.1 ± 0.4
500	66.1 ± 0.8	55.6 ± 0.6	1.2 ± 0.5
700	67.5 ± 1.4	67.1 ± 1.3	50.0 ± 2.8
1000	67.8 ± 1.8	67.3 ± 1.6	50.6 ± 2.3
1380	67.9 ± 1.2	67.3 ± 1.1	50.1 ± 2.9

**Table 4:** Per-class recall for the proposed 700-feature model (mean ± standard deviation across LOSO folds, %). Values are computed directly from the fold-level per-class recall recorded during the feature-selection sweep.

Modality	Model	Accuracy (%)	NP Recall (%)	LP Recall (%)	HP Recall (%)
HbO_2_	XGBoost	67.7 ± 7.1	100.0 ± 0.0	52.7 ± 17.5	50.4 ± 15.8
HHb	RF	69.6 ± 6.9	97.8 ± 3.7	58.3 ± 15.0	52.7 ± 13.2
HbT	SVM	69.5 ± 7.3	100.0 ± 0.0	53.3 ± 18.2	55.1 ± 16.4

**Table 5:** Exploratory analysis: top five most stable features among the strongest-ranked (top 10) mutual-information features across 65 LOSO folds. For interpretability only; the proposed model uses 700 features. Count: number of folds in which the feature was in the top 10; percentage: proportion of folds (count/65).

Modality	Feature	Count	Percentage
HbO_2_	granger_ch05_to_ch20	63	96.9
	granger_ch18_to_ch21	41	63.1
	granger_ch14_to_ch20	41	63.1
	granger_ch14_to_ch21	40	61.5
	granger_ch18_to_ch20	35	53.8
HHb	granger_ch06_to_ch19	42	64.6
	granger_ch05_to_ch19	35	53.8
	granger_ch02_to_ch19	31	47.7
	granger_ch17_to_ch19	28	43.1
	granger_ch03_to_ch05	24	36.9
HbT	granger_ch17_to_ch21	40	61.5
	granger_ch07_to_ch10	39	60.0
	granger_ch19_to_ch08	22	33.8
	granger_ch14_to_ch10	21	32.3
	granger_ch23_to_ch10	20	30.8

**Table 6:** Comparison with prior fNIRS pain studies. Studies on the AI4PAIN dataset (65 subjects) are marked with ^a^.

Study	N	Features	Model	Results (Acc.)
Pourshoghi et al. [33]	19	B-spline (15)	SVM	Bin: 94%
Lopez-Martinez et al. [34]	20	B-spline (10)	MT-MKL	Bin: 80%
Lopez-Martinez et al. [35]	43	Wavelet	HBLR	Bin: 81%
Fernandez Rojas et al. [36]	18	T, F, W (25)	SVM	Multi: 94.17%
Fernandez Rojas et al. [63]	18	—	Bi-LSTM	Multi: 90.6%
Fernandez Rojas et al. [38]	18	—	CNN-LSTM	Multi: 91.2%
Khan et al. [37]	30	Statistical	SVM	Multi: 68.51%
Khan et al. [64] ^a^	65	ETEP	ENS	Bin: 91.41%; Multi: 68.20%
AI4PAIN [57] ^a^	65	Statistical	SVM	Multi: 43.20%
Ours ^a^	65	Brain connectivity (700)	RF, SVM, XGBoost	Multi: 69.6%

## Data Availability

The data presented in this study are available on request from the corresponding author.
